# Effect of Different Aging Methods on the Formation of Aroma Volatiles in Beef Strip Loins

**DOI:** 10.3390/foods10010146

**Published:** 2021-01-12

**Authors:** Dongheon Lee, Hyun Jung Lee, Ji Won Yoon, Minsu Kim, Cheorun Jo

**Affiliations:** 1 Center for Food and Bioconvergence, Department of Agricultural Biotechnology, and Research Institute of Agriculture and Life Science, Seoul National University, Seoul 08826, Korea; sptails@snu.ac.kr (D.L.); leehj0113@gmail.com (H.J.L.); ony0114@gmail.com (J.W.Y.); hoptop@snu.ac.kr (M.K.); 2 Institute of Green Bio Science and Technology, Seoul National University, Pyeongchang 25354, Korea

**Keywords:** dry aging, wet aging, aroma volatiles, aging periods

## Abstract

This study investigated the effects of different aging methods on the changes in the concentrations of aroma volatiles of beef. One half (*n* = 15) of the beef strip loins were dry-aged, and the other half were wet-aged, and both aging processes continued for 28 days. The aroma volatiles from dry- and wet-aged samples were analyzed at seven-day intervals (*n* = 3 for each aging period). As the aging period increased, dry-aged beef showed higher concentrations of volatile compounds than those in wet-aged beef (*p* < 0.05). Most changes in the concentrations of aroma volatiles of dry-aged beef were associated with propanal, 2-methylbutanal, 2-methylpropanal, 1-butanamine, trimethylamine, 2-methyl-2-propanethiol, and ethyl propanoate, which were mainly produced by lipid oxidation and/or microbial activity (e.g., proteolysis and lipolysis) during the dry aging period. Therefore, we suggest that the differences in aroma between dry- and wet-aged beef could result from increased lipid oxidation and microbial activity in dry-aged beef possibly owing to its ambient exposure to oxygen.

## 1. Introduction

Aging is the process of storing meat in a controlled environment for a certain period to increase the palatability of meat [1]. Aging can be especially effective for beef with low consumer preference as value addition. There are two forms of aging: wet and dry [2]. In wet aging, meat is vacuum-packaged and stored in a refrigerated condition [1]. On the other hand, dry aging involves holding the meat unpacked in the open air [3]. Dry- and wet-aged beef are under different environmental factors, i.e., dry-aged beef is influenced by not only temperature but also relative humidity and air flow, and is exposed to oxygen, while wet-aged beef is in anaerobic conditions. Due to the different aging conditions, many studies reported different physicochemical attributes between dry- and wet-aged beef [2,4]. Furthermore, the flavor of dry- and wet-aged beef is known to be discriminable. In general, dry-aged beef has more beefy, roasted, and nutty flavor, while wet-aged beef has a more intense sour, metallic, and bloody flavor [4].

Flavor is important for determining eating quality of meat, which affects consumer preferences [5]. Flavor is defined as the combination of taste and aroma and is attributed to different flavor compounds [6]. Flavor compounds such as amino acids, sugars, organic acids, and inorganic salts can contribute to the five different taste sensations and participate in aroma development of beef under heating condition [7]. Therefore, in order to explain the different flavor of dry- and wet-aged beef, several studies compared the flavor compounds of dry- and wet-aged beef [2,8]. Kim et al. [2] reported that dry-aged beef had significantly higher amounts of free amino acids, including glutamic acid and aspartic acid, than those of wet-aged beef. In addition, Lee et al. [8] confirmed that dry aging of beef for 28 days showed significantly higher content of free amino acids and reducing sugars than those in wet aging of beef.

As for the aroma, Watanabe et al. [9] and Yang et al. [10] reported that wet aging had an effect on increasing the levels of volatile compounds such as aldehydes and furans as the aging period increased. However, there is little information about aroma volatiles in dry-aged beef, which could be important to understand the characteristics of dry-aged beef flavor and its contributors. From this point of view, the changes in the concentrations of volatile compounds of beef with different aging methods may provide valuable information to elucidate the effect of dry aging on its desirable flavor. Considering the description of flavor in dry- and wet-aged beef from the literature [1,4], we hypothesized that each aging method (dry and wet aging) may have different effects on the formation of aroma volatiles in beef, leading to differences in the change of volatile patterns during the aging process. Therefore, we analyzed the volatile compounds in dry- and wet-aged beef during 28 days of aging.

## 2. Materials and Methods

### 2.1. Raw Material and Aging Process

In this study, 30 beef strip loins (*M. longissimus lumborum*) from both sides of 15 carcasses (21-month-old Holstein steers, quality grade 2) were purchased at 48 h post-mortem and transferred to a laboratory. The quality grade of the samples was based on the Korean beef grading system [11]. Approximately 500 g of lean meat was cut from each strip loin, and its initial pH (5.52 ± 0.01) was measured before aging (SevenGo, Mettler-Toledo, Schwerzenbach, Switzerland). Then, the samples were allocated to dry or wet aging randomly (*n* = 15 for each aging method). For wet aging, the samples were vacuum packaged (HFV-600L, Hankook Fujee Machinery Co., Ltd., Hwaseong, Korea) in low density polyethylene/nylon bags (O_2_ permeability of 2 mL/m^2^/d at 0 °C; 0.09 mm thickness; Sunkyung Co., Ltd., Seoul, Korea) and stored at 4 °C, while dry aging was processed at 4 °C, relative humidity of 75%, and air flow velocity of 2.5 m/s without any packaging. Both dry and wet aging processes continued for 28 days, and the samples from each group were collected on day 0, 7, 14, 21, and 28 (*n* = 3 for each aging period). Before sampling, the crust of dry-aged beef (approximately 0.5 cm from surface) was trimmed off. The beef samples were vacuum packaged and frozen at −70 °C until the volatile compound analysis.

### 2.2. Volatile Compound Analysis

Volatile compounds in dry- and wet-aged beef were analyzed by electronic nose (Heracles II, Alpha MOS, Toulouse, France) [8]. The frozen samples were thawed for 12 h at 4 °C and ground using a meat grinder (MG510, Kenwood, Hampshire, UK). Then, each sample (5 g) was weighed in a 20 mL vial and cooked for 10 min at 80 °C to obtain the volatile compounds without possible loss in sampling process after cooking. Then, the volatiles were injected into an electronic nose equipped with dual columns of MXT-5 and MXT-1701 (10 m × 180 μm × 0.4 μm; length × diameter × thickness) (Restek, Bellefonte, PA, USA). The analytical conditions for volatile compounds are in Table 1. Each peak was integrated and identified using retention time and relevance index indicating the percentage of matching probability, based on the comparison of Kovats retention index of the detected compound and the Kovats retention indices of known compounds from the AroChemBase library (Alpha MOS).

### 2.3. Mold Distribution

Mold distribution on the surface of dry-aged beef was analyzed using photographic imaging software (Adobe Photoshop CC 2015, Adobe, CA, USA) according to Oh et al. [12]. A photo of dry-aged beef illuminated with an LED light (MS-273, Myung Sung, Suwon, Korea) at 108 lx was taken (CMOS 16.0 MP, Samsung Co., Suwon, Korea). Mold distribution was measured by calculating the proportion of pixels over 128 levels in blue channel.

### 2.4. Free Fatty Acids

Free fatty acid contents in dry- and wet-aged beef were assessed by the method of Lee et al. [3]. Briefly, 1 g of lipid was put into the test tube with 1 mL of chloroform and an internal standard (1 mg of triundecanoate/mL isooctane). After removing triglycerides from the samples, free fatty acids were extracted using 2% acetic acid in diethyl ether. The extract was evaporated with nitrogen gas and heated at 85 °C for 10 min. After that, 2 mL of 14% boron trifluoride–methanol was put into the test tube for methylation and heated at the same condition. Then, 2 mL of isooctane and 1 mL of saturated sodium chloride were added into the test tube and centrifuged at 1573× *g* for 3 min (Continent 512R, Hanil Co. Ltd., Daejeon, Korea). The upper layer containing fatty acid methyl ester (FAME) was dehydrated with anhydrous sodium sulfate. FAME was analyzed using gas chromatography (HP 7890, Agilent Technologies, Santa Clara, CA, USA) with a DB-23 column (60 m × 250 μm × 0.25 μm; length × diameter × thickness) (Supelco, Bellefonte, PA, USA). Each FAME was identified by comparing the retention time of external standards (Supelco^®^ 37 Component FAME mix, Sigma-Aldrich, St. Louis, MO, USA).

### 2.5. Statistical Analysis

All samples for volatile analysis were triplicated, and statistical analysis was performed using SAS 9.4 program (SAS Institute Inc., Cary, NC, USA). The effects of different aging methods on the aroma pattern in volatile changes of beef strip loins were evaluated by two-way analysis of variance, and the model was analyzed with the fixed factors (aging method and aging period) and the random factors (carcass and side of the carcass). Mean values with standard error of the means were reported, and their significant differences were determined by the Student–Newman–Keuls multiple comparison test at a significance level of 0.05. Principal component analysis (PCA) was performed to discriminate aroma patterns in dry- and wet-aged beef by their volatile compounds. Pearson correlation coefficients between volatile compounds, mold distribution and unsaturated fatty acids of dry- and wet-aged beef were analyzed.

## 3. Results

### 3.1. Volatile Profiling of Aged Beef

A total of 37 volatile compounds in dry- and wet-aged beef were identified during 28 days of aging period (Table 2). They were assigned to the following chemical groups: aldehydes, furans, and ketones (*n* = 6); N-containing compounds (*n* = 4); S-containing compounds (*n* = 3); alcohols (*n* = 4); hydrocarbons, esters, and acids (*n* = 13); and others (*n* = 7). There was an aging method × aging period interaction for all identified volatile compounds (*p* < 0.01 for methyl 2-butenoate and *p* < 0.0001 for other compounds) except 4-nonanol (*p* = 0.22).

#### 3.1.1. Aldehydes, Furans and Ketones

Aldehyde contents increased in dry-aged beef, while a decreasing trend during wet aging was observed, with some fluctuations as the aging duration increased (Table 3). From day 14, the dry-aged beef had a significantly higher concentration of total aldehydes compared to those in wet-aged beef. The changes in the contents of aldehydes were affected mainly by propanal, which was predominant in both aging conditions but much higher in dry aging. Dry-aged beef also had significantly higher abundance of 2-methylbutanal than that in wet-aged beef during the whole aging period, and the concentration of 2-methylbutanal was the highest at day 28. The 2-methylpropanal level significantly increased at day 28 in dry-aged beef, whereas it could not be observed in wet-aged beef from day 14. (E, E)-2, 4-hexadienal content decreased significantly after 28 days of both dry and wet aging, although the concentrations were relatively small compared to other aldehydes. Propanal is considered an indicator of lipid oxidation [5]. Thus, the difference in propanal content between dry- and wet-aged beef, especially after 14 days, might result from the different susceptibility in the lipid oxidation process. Lipid oxidation is restrained during wet aging, because vacuum packaging prevents the exposure of meat to oxygen [29]. Kahraman and Gurbuz [30] reported that 2-thiobarbituric acid reactive substance values of dry-aged beef were significantly higher than those of wet-aged beef from 14 days of aging, indicating that oxidation of lipid occurred more actively during dry aging. The compounds 2-methylbutanal and 2-methylpropanal can be formed by Strecker degradation of isoleucine and valine, respectively [31]. Kim et al. [32] observed that the levels of tryptophan, phenylalanine, valine, tyrosine, glutamate, isoleucine, and leucine were significantly higher in three-week dry-aged beef compared to wet-aged beef. Lee et al. [3,8] also showed that the concentration of 18 free amino acids, including isoleucine and valine, was significantly higher in dry-aged beef than those in wet-aged beef, mainly due to microbial proteolysis. Therefore, we suggest that higher concentrations of 2-methylbutanal and 2-methylpropanal in dry-aged beef than those in wet-aged beef could be attributed to the higher concentration of isoleucine and valine due to the increased microbial activity during dry aging [8]. It was reported that microbial metabolism favored the production of branched aldehydes in fermented meat products [33]. Aldehydes contribute largely to beef aroma with sweet, floral, salty, and cheesy notes, because they have low odor thresholds [28,34]. Hence, differences in aldehyde content between differently aged beef could play an important role in creating a characteristic aroma.

Furans are odor-active volatiles formed by the oxidation of fatty acids [28]. As shown in Table 3, both types of aging methods increased the concentration of tetrahydrofuran with the increase in aging time (*p* < 0.05). Especially, dry-aged beef showed a significantly higher concentration of tetrahydrofuran than that in wet-aged beef after 14 days of aging.

The compound 3-heptanone was only ketone detected in the experiment. Generally, ketones are known as lipid-oxidation products with low odor thresholds [31]. The concentration of 3-heptanone increased after dry aging (*p* < 0.05), and it was higher at days 14 and 21 than in any other periods. On the other hand, it significantly decreased in wet-aged beef at day 7 and disappeared at day 14 and 21. It was detected at day 28 and was significantly lower compared to that in unaged beef.

#### 3.1.2. N-Containing Compounds

Total N-containing compounds were mostly higher in wet-aged beef than those in dry-aged beef until day 21 (Table 4). However, at the end of the aging period, dry-aged beef showed higher concentrations of N-containing compounds than those in wet-aged beef (*p* < 0.05). Except for ethenyl-dimethylpyrazine, the levels of these compounds dramatically increased at the late phase of dry aging (day 21 to 28). During that time, the concentration of 1-butanamine in dry-aged beef increased more than 23-fold. Similarly, the trimethylamine level increased approximately 16-fold from day 21 to 28. As for 2-pentylpyridine, it was detected only at day 28 in dry-aged beef. The formation of amine compounds is usually attributed to the degradation of amino acids due to the decarboxylase activity of microorganisms [35]. Trimethylamine can be produced from the reduction of trimethylamine oxide by microorganisms, and has been widely used for the assessment of microbial activity [14]. The interaction of 2, 4-decadienal with either ammonia or α-amino group of amino acids is believed to form 2-pentylpyridine [7]. Finally, pyrazines result from the Maillard reaction [5]. Considering the possible origins of the N-containing compounds, it seemed that proteolysis and degradation of amino acids might be the main contributors to the increase in the concentration of these products. Proteolysis is influenced by the action of muscle endogenous enzymes and/or microorganism-origin enzymes [3]. Muscle endogenous proteolytic enzymes are responsible for meat tenderization at the early period of aging; however, their activities decrease as aging duration increases [1]. The activity of aminopeptidases C and H, which could contribute to the increase in the amount of free amino acids during aging, was the highest at day 4; however, it decreased afterwards and was maintained until day 50 [36]. In this regard, protein degradation at the late period might be related more to microbial enzyme activity than muscle endogenous enzymes [8]. Lee et al. [8] found that the levels of free amino acids and trimethylamine were significantly higher in dry-aged beef at day 28 compared to those in wet-aged beef, indicating that growth of mold and/or yeast on the surface of dry-aged beef might result in further proteolysis. Altogether, change in the concentrations of N-containing compounds at the late phase of aging were highly noticeable, especially in dry-aged beef, and this observation was likely to be associated with different microbial enzyme activities. Differences in the concentration of N-containing compounds in dry- and wet-aged beef could discriminate their aroma characteristics; these compounds are the most important flavor precursors for meaty or beef flavors, with very low odor detection thresholds [6,10].

#### 3.1.3. S-Containing Compounds

Overall, S-containing compound levels increased with time in both aging processes (Table 5). Those concentrations were mostly higher in dry-aged beef from day 14 compared to those in wet-aged beef. The level of 2-methyl-2-propanethiol showed a tendency to increase during dry aging, especially from day 21 to 28. An exception was carbon disulfide at day 28, which was more abundant in wet-aged beef. Dimethyl trisulfide generally decreased in both dry- and wet-aged beef, and, eventually, no difference was found between them at the end of the aging period (*p* < 0.05). S-containing compounds originate from the degradation of S-containing amino acids such as methionine, cysteine, and cystine [27]. Carbon disulfide and 2-propanethiol can be produced via the Strecker degradation of S-containing amino acids [37]. Dimethyl trisulfide is particularly related to methionine degradation [38]. Differences in S-containing compounds between dry- and wet-aged beef might also result from the different occurrence of proteolysis during aging.

#### 3.1.4. Alcohols

Total alcohol contents in dry-aged beef significantly increased with the increase in aging period, whereas those in wet-aged beef decreased from the beginning and then increased after day 14 (Table 6). Therefore, from the early phase of aging period, dry-aged beef showed significantly higher alcohol contents than those in wet-aged beef. The increase in alcohol levels in dry-aged beef was mostly attributed to the increase in 2-butanol concentration. It significantly increased during 28 days of dry aging, except for day 7. Wet-aged beef also showed an increase in 2-butanol concentration with the increase in aging period. However, its change was relatively lower than that in dry aging, resulting in a significantly lower concentration than that in dry aging from day 21. During 28 days of aging period, the concentration of 1-methoxy-2-propanol in dry-aged beef peaked at day 14 and significantly decreased thereafter, but it was still higher than that in wet-aged beef, which gradually decreased and then increased at day 28. Moreover, 4-methyl-1-hexanol was present only when the beef was dry-aged for 14 days or longer. The alcohol 4-nonanol appeared at an earlier time in dry-aged beef than that in wet-aged beef, although no significant differences were found from day 21. Unlike straight-chain alcohols, which generally result from the oxidation of unsaturated fatty acids, 2-butanol and branched-chain alcohols with low molecular weight are produced by microbial fermentation [31]. The significant differences in the levels of 2-butanol, 1-methoxy-2-propanol, and a branched-chain alcohol, 4-methyl-1-hexanol, in dry- and wet-aged beef might imply the effect of different microbial activities on the aroma volatiles of aged beef.

#### 3.1.5. Hydrocarbons, Esters, and Acids

We observed a significant increase in total hydrocarbons in aged beef, regardless of the aging method (Table 7). Total hydrocarbon levels were significantly higher in dry-aged beef than those in wet-aged beef at days 14 and 21 of aging. When aging duration reached 28 days, however, total hydrocarbon levels were more abundant in wet-aged beef compared to those in beef that had been dry-aged. Levels of 2, 2-dichloropropane and heptane significantly increased during wet aging period and, by the last day of aging, the concentrations of the compounds in wet-aged beef were significantly higher than those in dry-aged beef. On the contrary, butane and octane contents were significantly higher in dry-aged beef than those in wet-aged beef during the aging process. The compounds 3- and 4-methyldecane and ethylcyclopentane were detected in dry-aged beef only. When compared to the changes in hydrocarbons in dry-aged beef, the higher concentrations in wet-aged beef were not expected because they were believed to be derived mainly from the autoxidation of lipids [38]. As for wet-aged beef, Ma et al. [39] stated that 21 days of postmortem storage hardly affected the changes in levels of volatile compounds, and assumed that lipid oxidation progressed slowly up to three weeks under vacuum conditions. It is hard to explain the significantly higher concentration of 2, 2-dichloropropane in wet-aged beef than that in dry-aged beef. Nonetheless, this would not be the main factor for the distinctive aroma of dry- or wet-aged beef, because hydrocarbons have limited effects on the flavor of meat due to their high odor detection thresholds [40]. Meanwhile, 3- and 4-methyldecane, which appeared only in dry-aged beef, could have been generated by the secondary degradation of triglycerides by the activity of surface molds, possibly indicating the differences in microflora in dry- and wet-aged beef [34].

After 28 days of aging, the concentrations of esters increased in dry-aged beef and decreased in wet-aged beef (*p* < 0.05). During dry aging, a significant increase in ethyl propanoate level was detected from day 14. Moreover, an approximately six-fold increase in its concentration at day 28 compared to day 0 was observed. In contrast, ethyl propanoate content was significantly lower in wet-aged beef from day 7 to 21 compared to that in unaged beef. Methyl 2-butenoate was present only in dry-aged beef from day 14. Concentrations of propyl propanoate in both dry- and wet-aged beef showed lower values than those in unaged beef, and the content was higher in wet-aged beef at day 28 compared to that in dry-aged beef (*p* < 0.05). Finally, methyl 2-methylbutanoate levels showed significant changes in both dry- and wet-aged beef until day 21, but no significant difference was found between dry- and wet-aged beef at the last day of aging. Esters are produced by the esterification reaction of alcohols and acids and are related to the activity of microbial esterase [27,28]. Bruna et al. [33] reported that dry sausages inoculated with *Penicillium aurantiogriseum* had higher levels of esters, while sausages without inoculation had very few ester compounds present. Corral et al. [41] found that *Debaryomyces hansenii* inoculated into the sausages was responsible for the increase in the levels of ester compounds. Thus, differences in ester contents in dry- and wet-aged beef might indicate differences in metabolic activity of microorganisms affected by different aging methods.

As for acids, significantly higher concentrations of acids were observed in dry-aged beef after 14 days of aging compared to those in wet-aged beef. Generally, acids increased during dry aging, while they significantly decreased after wet aging. Moreover, 2-methylpropanoic acid was found only in dry-aged beef from day 14, and it decreased at day 28 (*p* < 0.05). Hexanoic acid was not detected in dry-aged beef at day 7, but its level was significantly higher in dry-aged beef after 14 days compared to that in wet-aged beef. Hexanoic acid can result from lipid oxidation, and 2-methylpropanoic acid can result from the oxidation of 2-methylpropanal [31]. In particular, Casaburi et al. [27] stated that branched-chain fatty acids such as 2- and 3-methylbutanoic acids were observed in aerobically-stored meat, not in vacuum-packaged meat. These compounds accounted for only a small portion of volatile contents in both dry- and wet-aged beef. Nevertheless, acids are regarded as important compounds, which can be used as substrates for the production of esters, strongly affecting the aroma of beef products [38].

### 3.2. Volatile Patterns of Aged Beef

**Figure 1:**
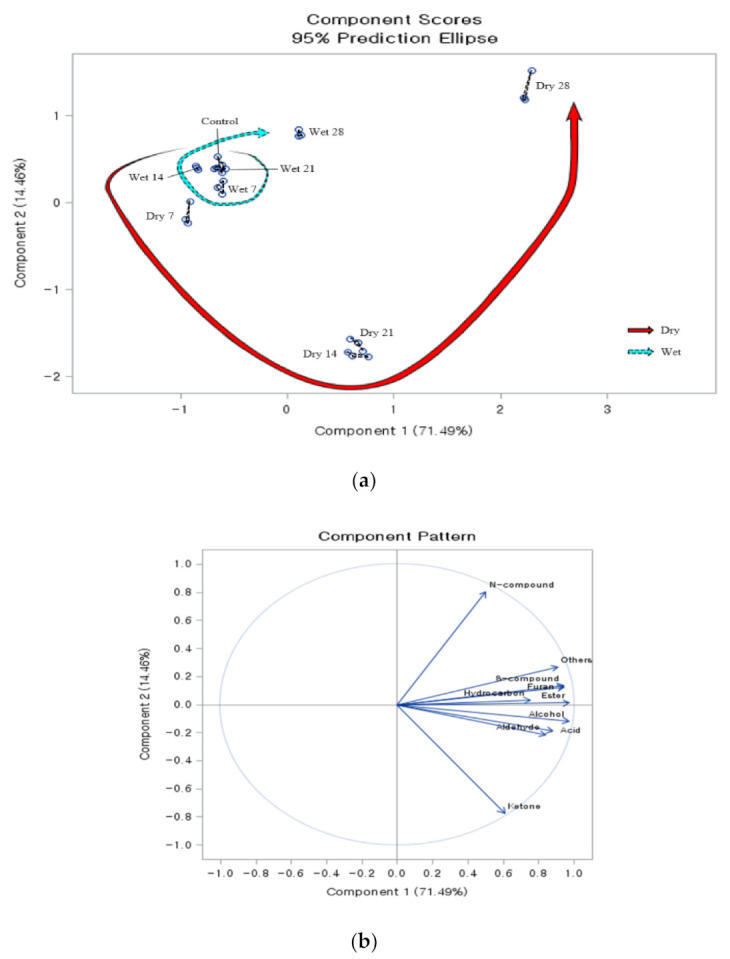
(**a**) Principal component analysis score plot; (**b**) the loading plot for the changes in patterns of aroma volatiles of beef during aging with different aging methods. Red solid-line arrow illustrates the change in aroma patterns in dry-aged beef, and blue dotted-line arrow indicates the change in aroma patterns in wet-aged beef during aging. Unaged beef (control), dry-aged beef (dry), wet-aged beef (wet), N-containing compound (N-compound), and S-containing compound (S-compound).

The patterns of aroma volatiles in beef sirloin changed with different trends in dry or wet aging (Figure 1a). For wet-aged beef, similar volatile patterns were observed until day 21, and only day 28 led to relatively distinct volatile patterns compared to those in unaged beef. The effect of wet aging on the development of volatile compounds is controversial. Watanabe et al. [9] observed that wet aging for 30 days significantly increased the levels of oxygen, nitrogen, and sulfur heterocyclic compounds, and concluded that wet aging could affect the flavor of beef. Similarly, Yang et al. [10] found that the levels of volatile compounds (e.g., aldehydes, alkanes, pyrazines, and furans) increased in beef after 14 days of wet aging. In contrast, several studies have documented that a wet aging duration of 2–3 weeks is not enough for the production of additional volatile compounds [39,40]. In this study, the differentiation in volatile patterns of wet-aged beef was affected mainly by the increase in 2, 2-dichloropropane, carbon disulfide, and tetrahydrofuran levels at the late phase of wet aging (Table 3, Table 5 and Table 7). However, when considering the high odor thresholds (Table 2) of 2, 2-dichloropropane, carbon disulfide, and tetrahydrofuran [18], the aroma of wet-aged beef may not be unique, which is in accordance with Jin and Yim [1].

On the contrary, diverse volatile patterns were observed in beef during dry aging (Figure 1a). From day 14, the aroma pattern of dry-aged beef could be differentiated from those of wet- or unaged beef. Similarly, Lee et al. [8] mentioned that the dry-aged flavor began to be perceived from day 14 to 21 of the aging period, and umami flavors could be intensified by extended aging. We observed an overall increase in the levels of volatile compounds in dry-aged beef, which were much higher than those in wet-aged beef. Lipid oxidation may be an important factor for the development of dry-aged flavor, because a large proportion of volatile compounds (e.g., propanal, hydrocarbons, furans, and ketones) derive from lipid oxidation. Among them, aldehydes are known as low odor threshold products, and a higher concentration of propanal in dry-aged beef may be responsible for the dry-aged flavor. Additionally, the PCA loading plot (Figure 1b) showed that 3-heptanone might be relevant to the characteristic volatile pattern from day 14 to 21, when its concentration was higher than in other aging periods. Due to 3-heptanone showing relatively low odor threshold values compared to other lipid oxidation-derived products like most hydrocarbons (Table 2), it might be regarded as an important volatile compound among the lipid oxidation-derived products in dry-aged beef. Moreover, the changes in the concentrations of N-containing compounds may be the key for the characteristics of aroma volatiles in dry-aged beef at day 28, indicating the importance of microbial activity in the formation of the unique dry-aged flavor. Given the low odor threshold values of trimethylamine (Table 2), it might be important for dry-aged flavor that the significant increase in trimethylamine was observed in dry-aged beef at day 28. This finding suggests the importance of microbial activity in the formation of the unique dry-aged flavor. Earlier studies have also reported noticeable changes in the levels of flavor compounds at the late phase of dry aging (from day 21 to 28). Lee et al. [8] observed significant increases in levels of flavor compounds of dry-aged beef between day 21 and 28, and explained that further proteolysis and lipolysis associated with microorganisms (especially mold and/or yeast) could develop aroma compounds of dry-aged beef. Oh et al. [42] observed that dry-aged beef inoculated with *Pilaira anomala* had higher oleic, palmitic, and stearic acid content at day 21, while inoculation with *D. hansenii* resulted in higher amounts of free amino acids at day 28. Finally, the contribution of 2-methylpropanal, 2-methylbutanal, 2-methyl-2-propanethiol, ethyl propanoate, and possibly 2-methylpropanoic acid to dry-aged flavor should be noted when their low odor threshold values (Table 2) and concentration differences between dry- and wet-aged beef are taken account (Table 3, Table 5 and Table 7). Particularly, the result that at day 14 of aging or more, 2-methylpropanal and 2-methylpropanoic acid only existed in dry-aged beef indicates the potential key volatile compounds. The rapid increase in the levels of these amino acid-degradation products (e.g., 2-methylbutanal, 2-methylpropanal, and 2-methyl-2-propanthiol) may have resulted from the increase in levels of flavor precursors by microbial metabolism at the late phase of dry aging. Although other compounds such as 2-butanol and tetrahydrofuran showed significantly higher concentrations in dry-aged beef compared to wet-aged beef, for their relatively high odor threshold values, they might act as minor contributors for dry-aged flavor.

### 3.3. Correlation Analysis

It was observed that most lipid oxidation-derived volatile compounds were positively correlated with unsaturated fatty acids in dry-aged beef (Table 8). Especially, there was a strong correlation between aldehydes and unsaturated fatty acids when the beef was dry-aged, mainly influenced by the increase in propanal, which was the most abundant compound in dry-aged beef (Table 3). The increase in mold on the surface of dry-aged beef also had positive correlations with all volatile compound groups, indicating the importance of microbial activity for the formation/increase in aroma volatile compounds at the late phase of dry aging period.

As we hypothesized, dry and wet aging led to distinct volatile composition and patterns of beef, showing the different effect on the formation of aroma volatiles. Lipid oxidation and microbial activity might play a role as flavor contributors in aged beef, and their influences were more noticeable in dry-aged beef than wet-aged beef.

## 4. Conclusions

Dry-aged beef showed significantly higher concentrations of volatile compounds, with more distinctive changes than those in wet-aged beef. This was mainly attributed to: (i) propanal known to be generated from the oxidation of lipids; and (ii) 2-methylbutanal, 2-methylpropanal, 1-butanamine, trimethylamine, 2-methylpropanthiol, and ethyl propanoate, possibly derived from the metabolism of microorganisms. Based on the results, the formation of aroma volatiles responsible for the unique flavor in dry-aged beef, clearly separated from wet-aged counterpart, may be induced by lipid oxidation and microbial activity.

## Figures and Tables

**Table 1:** Analytical conditions of electronic nose for volatile compounds in dry- and wet-aged beef strip loins during 28 days of aging.

Parameter		Condition
Headspace generation	Incubation temperature	80 °C
	Incubation time	10 min
Trap	Initial temperature	40 °C
	Split	10 mL/min
	Trapping duration	30 s
	Final temperature	240 °C
Injector	Carrier gas	Hydrogen
	Injected volume	5 mL
	Injected speed	250 µL/s
	Injector temperature	200 °C
Column	Column temperature	40 °C for 5 s
		Increased at 0.5 °C/s, held for 5 s at 150 °C
		Increased at 5 °C/s, held for 30 s at 260 °C
	Acquisition duration	282 s
Detector	Type	Flame ionized detector (dual)

**Table 2:** Identified volatile compounds in dry- or wet-aged beef strip loins during 28 days of aging.

No.	Volatile Compound	RT ^1^	RI ^2^	Aroma Description	Odor Threshold (ppm)
*Aldehydes, furans, and ketones*					
1	(E, E)-2, 4-Hexadienal	116.86	81.10	Citrus, floral, green, spicy, sweet	94.8 [13]
2	2-Methylbutanal	28.30	92.02	Ethereal, nutty, sweet [14]	1 [14]
3	2-Methylpropanal	16.39	51.36	Camphor, green, malty, pungent, sharp [15]	0.7 [13]
4	Propanal	14.13	65.50	Almond, cherry, green, fruity [14]	25.1 [14]
5	Tetrahydrofuran	21.72	93.82	Aromatic, burnt, fruity, sulfurous, sweet	92-61,000 [16]
6	3-Heptanone	87.14	90.67	Fatty, fruity, green, spicy, sweet	140 [17]
*N-containing compounds*					
1	1-Butanamine	25.03	85.38	Ammoniacal, fishy	170 [18]
2	Ethenyl-dimethylpyrazine	147.23	93.75	Earthy, musty	no reference
3	2-Pentylpyridine	108.94	84.18	Fatty, green, mushroom, pepper, tallowy	5 [19]
4	Trimethylamine	11.10	74.73	Ammoniacal, fishy, fruity, oily, pungent, rancid, sweaty	2.4 [14]
*S-containing compounds*					
1	2-Methyl-2-propanethiol	17.99	88.64	Sulfurous	0.33 [20]
2	Carbon disulfide	17.30	71.60	Burnt, cabbage, fruity, sulfurous [15]	210 [18]
3	Dimethyl trisulfide	101.67	85.46	Alliaceous, cabbage, fishy, meaty, onion, sulfurous	0.1 [14]
*Alcohols*					
1	1-Methoxy-2-propanol	34.76	76.18	Mild	839-33,000 [16]
2	2-Butanol	28.35	56.68	Pleasant, strong, sweet, wine	220 [18]
3	4-Methyl-1-hexanol	97.21	92.13	Grassy, sweaty, nutty, oily, roasty	2000 [21]
4	4-Nonanol	139.31	76.28	Sweet [21,22]	no reference
*Hydrocarbons, esters, and acids*					
1	2, 2-Dichloropropane	21.81	89.93	no reference	no reference
2	3-Methyldecane	134.73	82.85	Balsamic, mild, phenolic	no reference
3	4-Methyldecane	132.13	91.50	Fatty, fresh, waxy [23]	no reference
4	Butane	11.08	68.12	Faint	1,200,000 [18]
5	Ethylcyclopentane	38.58	76.25	Alkane, fruity, gasoline, sweet	no reference
6	Heptane	33.95	60.05	Floral, fruity, sweet	400,000 [24]
7	Octane	41.04	95.87	Alkane, fruity, sweet, fatty, solvent [15]	1700 [18]
8	Ethyl propanoate	31.10	86.29	Burnt, fermented, fruity, green, malty, nutty, sour	0.01 [25]
9	Methyl 2-methylbutanoate	44.63	90.84	Fatty, fruity, green	0.4 [26]
10	Methyl 2-butenoate	41.36	77.98	Blackcurrant, fruity	no reference
11	Propyl propanoate	58.53	90.15	Fruity, green, sweet	0.88 [25]
12	2-Methylpropanoic acid	49.12	77.12	Dairy, fatty, pungent, rancid, sour, sweaty	50 [17]
13	Hexanoic acid	110.28	91.42	Cheesy, fatty, pungent, rancid, sour, sweaty	3,000,000 [27]
*Others*					
1	1, 2, 4-Thiadiazole, 5-ethoxy-3-(trichloromethyl)-	248.10	87.34	Mild	no reference
2	Demeton-O	264.10	59.09	no reference	no reference
3	Diisopropyl ether	16.83	89.64	Ethereal	no reference
4	Ethyl chloride	13.93	92.29	Ethereal, pungent	3800–379,000 [16]
5	Limonene	125.69	78.19	Citrus, fruity, minty	38 [18]
6	P-cymene	121.27	93.22	Citrus, fruity, herbaceous, pleasant, solvent, spicy, sweet	120 [28]
7	Perfluorononane	9.25	87.80	no reference	no reference

^1^ RT, retention time; ^2^ RI, relevance index indicating the percentage of matching probability based on the comparison of Kovats retention index of the detected compound and the Kovats retention indices of known compounds from the AroChemBase library.

**Table 3:** Peak area of aldehydes, furans, and ketones in beef during aging with different aging methods.

Compound	Aging Method	Aging Period (d)					SEM ^1^
		0	7	14	21	28	
*Aldehyde*							
(E, E)-2, 4-Hexadienal	Dry	376 ^a^	137 ^bx^	99 ^c^	113 ^cy^	153 ^bx^	6.5
	Wet	376 ^a^	121 ^by^	94 ^c^	137 ^bx^	56 ^dy^	6.7
	SEM ^2^	10.5	2.9	8.8	4.1	2.5	
2-Methylbutanal	Dry	845 ^c^	742 ^cx^	2495 ^bx^	1667 ^cx^	4503 ^ax^	246.9
	Wet	845 ^b^	529 ^cy^	341 ^dy^	639 ^cy^	1827 ^ay^	47.8
	SEM ^2^	7.8	46.1	353.7	40.5	170.9	
2-Methylpropanal	Dry	506 ^b^	657 ^by^	864 ^bx^	1077 ^bx^	2458 ^ax^	309.1
	Wet	506 ^b^	821 ^ax^	nd ^cy^	nd ^cy^	nd ^cy^	7.3
	SEM ^2^	11.4	9.0	38.2	9.4	487.1	
Propanal	Dry	17,635 ^c^	9067 ^dy^	43,508 ^ax^	20,185 ^cx^	38,743 ^bx^	1139.6
	Wet	17,635 ^a^	14,111 ^bx^	16,915 ^ay^	12,344 ^cy^	14,394 ^by^	275.4
	SEM ^2^	300.0	663.7	676.5	838.4	1321.1	
Total	Dry	19,361 ^b^	10,603 ^cy^	46,966 ^ax^	23,042 ^bx^	45,857 ^ax^	1174.5
	Wet	19,361 ^a^	15,582 ^cx^	17,349 ^by^	13,119 ^dy^	16,277 ^cy^	313.7
	SEM ^2^	296.2	683.3	1027.0	841.9	1173.3	
*Furan*							
Tetrahydrofuran	Dry	292 ^e^	900 ^d^	2143 ^cx^	3731 ^bx^	6262 ^ax^	60.6
	Wet	292 ^e^	960 ^d^	1128 ^cy^	1399 ^by^	3068 ^ay^	21.4
	SEM ^2^	9.4	31.2	28.6	35.8	84.6	
*Ketone*							
3-Heptanone	Dry	104 ^e^	352 ^dx^	1237 ^bx^	1465 ^ax^	675 ^cx^	19.4
	Wet	104 ^a^	83 ^by^	nd ^dy^	nd ^dy^	60 ^cy^	3.6
	SEM ^2^	6.6	17.4	7.3	22.6	8.0	

^1^ Standard error of the mean (*n* = 15), ^2^ (*n* = 6); ^a–e^ Different letters within same row differ significantly (*p* < 0.05); ^x,y^ Different letters within same column differ significantly (*p* < 0.05); nd, not detected.

**Table 4:** Peak area of N-containing compounds in beef during aging with different aging methods.

Compound	Aging Method	Aging Period (d)					SEM ^1^
		0	7	14	21	28	
1-Butanamine	Dry	274 ^b^	72 ^by^	334 ^bx^	133 ^by^	3048 ^ax^	62.9
	Wet	274 ^a^	135 ^cx^	145 ^cy^	218 ^bx^	87 ^dy^	5.5
	SEM ^2^	11.4	7.6	4.8	10.9	98.2	
Ethenyl-dimethylpyrazine	Dry	443 ^b^	751 ^ax^	441 ^bx^	488 ^bx^	507 ^b^	41.9
	Wet	443 ^a^	357 ^by^	246 ^cy^	218 ^cy^	364 ^b^	10.1
	SEM ^2^	6.5	42.5	20.5	7.1	48.1	
2-Pentylpyridine	Dry	nd ^b^	nd ^b^	nd ^b^	nd ^by^	425 ^ax^	1.4
	Wet	nd ^c^	nd ^c^	nd ^c^	361 ^ax^	188 ^by^	8.4
	SEM ^2^	-	-	-	1.1	13.4	
Trimethylamine	Dry	11,806 ^b^	4728 ^c^	2045 ^dy^	1052 ^dy^	17,487 ^ax^	546.6
	Wet	11,806 ^a^	4924 ^d^	6727 ^cx^	6949 ^cx^	9019 ^by^	212.8
	SEM ^2^	110.4	611.2	145.3	232.9	631.7	
Total	Dry	12,523 ^b^	5551 ^c^	2820 ^dy^	1673 ^dy^	21,466 ^ax^	605.5
	Wet	12,523 ^a^	5415 ^d^	7117 ^cx^	7746 ^cx^	9658 ^by^	216.6
	SEM ^2^	127.4	601.9	154.1	220.4	763.5	

^1^ Standard error of the mean (*n* = 15), ^2^ (*n* = 6); ^a–d^ Different letters within same row differ significantly (*p* < 0.05); ^x,y^ Different letters within same column differ significantly (*p* < 0.05); nd, not detected.

**Table 5:** Peak area of S-containing compounds in beef during aging with different aging methods.

Compound	Aging Method	Aging Period (d)					SEM ^1^
		0	7	14	21	28	
2-Methyl-2-propanethiol	Dry	1025 ^d^	875 ^dy^	5342 ^bx^	2729 ^cx^	9680 ^ax^	142.8
	Wet	1025 ^c^	1644 ^ax^	1015 ^cy^	754 ^dy^	1477 ^by^	30.6
	SEM ^2^	41.2	24.0	193.9	76.1	87.7	
Carbon disulfide	Dry	1174 ^d^	758 ^dy^	4401 ^cx^	5267 ^bx^	8403 ^ay^	168.3
	Wet	1174 ^e^	3628 ^bx^	1523 ^dy^	1989 ^cy^	9787 ^ax^	62.7
	SEM ^2^	8.4	67.6	68.2	234.8	127.1	
Dimethyl trisulfide	Dry	122 ^a^	125 ^ay^	138 ^ax^	101 ^bx^	79 ^c^	4.7
	Wet	122 ^b^	133 ^ax^	79 ^cy^	59 ^ey^	66 ^d^	1.7
	SEM ^2^	2.7	1.3	4.1	4.5	4.1	
Total	Dry	2320 ^d^	1758 ^dy^	9882 ^bx^	8097 ^cx^	18,162 ^ax^	261.7
	Wet	2320 ^e^	5405 ^bx^	2616 ^dy^	2802 ^cy^	11,330 ^ay^	49.9
	SEM ^2^	32.5	63.0	223.2	300.5	179.7	

^1^ Standard error of the mean (*n* = 15), ^2^ (*n* = 6); ^a–e^ Different letters within same row differ significantly (*p* < 0.05); ^x,y^ Different letters within same column differ significantly (*p* < 0.05).

**Table 6:** Peak area of alcohols in beef during aging with different aging methods.

Compound	Aging Method	Aging Period (d)					SEM ^1^
		0	7	14	21	28	
1-Methoxy-2-propanol	Dry	749 ^d^	723 ^dx^	2532 ^ax^	2212 ^bx^	1515 ^cx^	36.8
	Wet	749 ^a^	291 ^by^	106 ^cy^	17 ^dy^	316 ^by^	20.7
	SEM ^2^	33.7	37.3	24.9	17.8	31.4	
2-Butanol	Dry	102 ^c^	nd ^dy^	891 ^c^	2005 ^bx^	5308 ^ax^	231.3
	Wet	102 ^d^	146 ^dx^	236 ^c^	402 ^by^	890 ^ay^	19.1
	SEM ^2^	3.9	4.2	280.7	41.5	232.5	
4-Methyl-1-hexanol	Dry	nd ^d^	nd ^d^	120 ^cx^	168 ^ax^	131 ^bx^	3.2
	Wet	nd	nd	nd ^y^	nd ^y^	nd ^y^	-
	SEM ^2^	-	-	2.2	1.9	4.2	
4-Nonanol	Dry	nd ^c^	nd ^c^	19 ^bx^	60 ^b^	135 ^a^	14.1
	Wet	nd ^c^	nd ^c^	nd ^cy^	60 ^b^	177 ^a^	11.0
	SEM ^2^	-	-	13.2	3.3	24.9	
Total	Dry	850 ^d^	723 ^dx^	3562 ^cx^	4444 ^bx^	7089 ^ax^	215.8
	Wet	850 ^b^	437 ^cy^	342 ^dy^	479 ^cy^	1382 ^ay^	28.2
	SEM ^2^	31.3	38.1	266.2	50.9	206.2	

^1^ Standard error of the mean (*n* = 15), ^2^ (*n* = 6); ^a–d^ Different letters within same row differ significantly (*p* < 0.05); ^x,y^ Different letters within same column differ significantly (*p* < 0.05); nd, not detected.

**Table 7:** Peak area of hydrocarbons, esters, and acids in beef during aging with different aging methods.

Compound	Aging Method	Aging Period (d)					SEM ^1^
		0	7	14	21	28	
*Hydrocarbon*							
2, 2-Dichloropropane	Dry	2604 ^e^	4274 ^d^	12,837 ^bx^	11,361 ^c^	15,398 ^ay^	319.2
	Wet	2604 ^c^	4931 ^c^	5041 ^cy^	7597 ^b^	20,477 ^ax^	659.1
	SEM ^2^	35.8	231.0	405.9	992.9	368.0	
3-Methyldecane	Dry	nd ^b^	nd ^b^	nd ^b^	nd ^b^	98 ^ax^	1.2
	Wet	nd	nd	nd	nd	nd ^y^	-
	SEM ^2^	-	-	-	-	1.9	
4-Methyldecane	Dry	nd ^b^	nd ^b^	nd ^b^	nd ^b^	149 ^ax^	1.7
	Wet	nd	nd	nd	nd	nd ^y^	-
	SEM ^2^	-	-	-	-	2.6	
Butane	Dry	251 ^d^	316 ^bx^	280 ^cx^	297 ^bcx^	715 ^ax^	8.5
	Wet	251 ^b^	203 ^dy^	233 ^cy^	251 ^by^	261 ^ay^	1.7
	SEM ^2^	1.1	2.7	0.6	2.9	13.1	
Ethylcyclopentane	Dry	nd ^c^	nd ^c^	799 ^bx^	1430 ^ax^	1372 ^ax^	48.9
	Wet	nd	nd	nd ^y^	nd ^y^	nd ^y^	-
	SEM ^2^	-	-	31.7	61.9	33.9	
Heptane	Dry	nd ^d^	nd ^d^	169 ^ax^	127 ^cy^	149 ^by^	6.3
	Wet	nd ^d^	nd ^d^	28 ^cy^	1370 ^bx^	1537 ^ax^	40.5
	SEM ^2^	-	-	20.5	35.4	50.2	
Octane	Dry	803 ^d^	937 ^cd^	998 ^cx^	1300 ^bx^	1791 ^ax^	44.0
	Wet	803 ^b^	844 ^b^	485 ^cy^	967 ^ay^	602 ^cy^	36.2
	SEM ^2^	60.5	44.3	18.0	14.9	44.0	
Total	Dry	3658 ^d^	5527 ^c^	15,083 ^bx^	14,515 ^bx^	19,671 ^ay^	340.0
	Wet	3658 ^c^	5978 ^c^	5787 ^cy^	10,184 ^by^	22,877 ^ax^	692.2
	SEM ^2^	58.2	265.9	426.9	1043.7	375.7	
*Ester*							
Ethyl propanoate	Dry	1009 ^c^	381 ^d^	2854 ^bx^	3087 ^bx^	6761 ^ax^	107.5
	Wet	1009 ^a^	327 ^b^	254 ^by^	298 ^by^	829 ^ay^	73.3
	SEM ^2^	154.2	74.8	25.8	35.1	105.0	
Methyl 2-methylbutanoate	Dry	326 ^b^	205 ^dy^	261 ^cx^	367 ^ax^	358 ^a^	7.6
	Wet	326 ^b^	234 ^dx^	202 ^ey^	280 ^cy^	372 ^a^	8.2
	SEM ^2^	13.2	6.7	4.2	5.6	6.6	
Methyl 2-butenoate	Dry	nd ^b^	nd ^b^	123 ^ax^	187 ^ax^	158 ^ax^	30.9
	Wet	nd	nd	nd ^y^	nd ^y^	nd ^y^	-
	SEM ^2^	-	-	3.8	5.9	48.3	
Propyl propanoate	Dry	3015 ^a^	80 ^ey^	715 ^bx^	322 ^dx^	471 ^cy^	15.1
	Wet	3015 ^a^	380 ^cx^	150 ^dy^	176 ^dy^	1183 ^bx^	20.5
	SEM ^2^	23.4	19.9	11.9	8.0	21.6	
Total	Dry	4349 ^b^	666 ^d^	3953 ^cx^	3964 ^cx^	7748 ^ax^	88.5
	Wet	4349 ^a^	941 ^c^	606 ^dy^	754 ^cdy^	2383 ^by^	65.8
	SEM ^2^	136.2	77.5	29.6	44.5	54.7	
*Acid*							
2-Methylpropanoic acid	Dry	nd ^c^	nd ^c^	87 ^ax^	82 ^ax^	64 ^bx^	1.9
	Wet	nd	nd	nd ^y^	nd ^y^	nd ^y^	-
	SEM ^2^	-	-	2.6	1.0	1.2	
Hexanoic acid	Dry	167 ^b^	nd ^cy^	165 ^bx^	140 ^bx^	248 ^ax^	9.5
	Wet	167 ^a^	99 ^bx^	nd ^cy^	108 ^by^	119 ^by^	8.7
	SEM ^2^	15.8	6.7	5.5	5.9	7.6	
Total	Dry	167 ^d^	nd ^ey^	252 ^bx^	222 ^cx^	311 ^ax^	8.9
	Wet	167 ^a^	99 ^bx^	nd ^cy^	108 ^by^	119 ^by^	8.7
	SEM ^2^	15.8	6.7	4.1	5.6	6.6	

^1^ Standard error of the mean (*n* = 15), ^2^ (*n* = 6); ^a–e^ Different letters within same row differ significantly (*p* < 0.05); ^x,y^ Different letters within same column differ significantly (*p* < 0.05); nd, not detected.

**Table 8:** Pearson correlation coefficient for volatile compounds in dry- and wet-aged beef with their mold distribution and unsaturated free fatty acid contents.

Chemical Group	Mold Distribution		Unsaturated Fatty Acids	
	Dry	Wet	Dry	Wet
Acid	0.63 *	-	0.83 **	ns
Alcohol	0.84 ***	-	0.61 *	ns
Aldehyde	0.59 *	-	0.84 ***	−0.71 **
Ester	0.81 **	-	0.67 **	ns
Furan and ketone	0.78 **	-	ns	ns
Hydrocarbon	0.71 **	-	0.63 *	ns
N-compound	0.80 **	-	ns	ns
S-compound	0.87 ***	-	0.62 *	ns

Mold distribution was conducted for dry-aged beef only, because no mold growth was shown in wet-aged beef; *, *p* < 0.05; **, *p* < 0.01; ***, *p* < 0.0001; ns, not significant (*p* > 0.05).

## Data Availability

Not applicable.
